# Correlation test to assess low-level processing of high-density oligonucleotide microarray data

**DOI:** 10.1186/1471-2105-6-80

**Published:** 2005-03-31

**Authors:** Alexander Ploner, Lance D Miller, Per Hall, Jonas Bergh, Yudi Pawitan

**Affiliations:** 1 Medical Epidemiology and Biostatistics, Karolinska Institutet, Stockholm, Sweden; 2 Genome Institute of Singapore, Singapore; 3 Department of Oncology and Pathology, Cancer Center Karolinska, Radiumhemmet, Karolinska Institutet and University Hospital, Stockholm

## Abstract

**Background:**

There are currently a number of competing techniques for low-level processing of oligonucleotide array data. The choice of technique has a profound effect on subsequent statistical analyses, but there is no method to assess whether a particular technique is appropriate for a specific data set, without reference to external data.

**Results:**

We analyzed coregulation between genes in order to detect insufficient normalization between arrays, where coregulation is measured in terms of statistical correlation. In a large collection of genes, a random pair of genes should have on average zero correlation, hence allowing a correlation test. For all data sets that we evaluated, and the three most commonly used low-level processing procedures including MAS5, RMA and MBEI, the housekeeping-gene normalization failed the test. For a real clinical data set, RMA and MBEI showed significant correlation for absent genes. We also found that a second round of normalization on the probe set level improved normalization significantly throughout.

**Conclusion:**

Previous evaluation of low-level processing in the literature has been limited to artificial spike-in and mixture data sets. In the absence of a known gold-standard, the correlation criterion allows us to assess the appropriateness of low-level processing of a specific data set and the success of normalization for subsets of genes.

## Background

The spread of microarray technology has made possible the routine and simultaneous measurement of expression profiles for tens of thousands of genes. In the case of photolithographically synthesized high-density oligonucleotide arrays as described in [1], the technology for hybridizing RNA on chips and quantitating fluoresence-intensity data has been highly standardized and automated. The results are then related to the biology of interest, both through exploratory methods (e.g. [2]) and a large and growing number of sophisticated prediction and classification algorithms (e.g. [3]). Yet the very first step on which these procedures rely is still open to discussion: the derivation of a numerical summary value that is both representative of a gene's relative expression level and reasonably free of technical variation, summarily referred to as low-level analysis.

The need for a summary function is due to the setup of high-density oligonucleotide arrays, where each gene is probed by a set of paired oligonucleotides: one of each pair matches the target sequence on the probed gene perfectly (perfect match or PM oligo), the other has one altered central base-pair (mismatch or MM oligo), where the MMs serve to establish a reference for non-specific hybridisation. While the full set of PMs has been used successfully for detecting differential expression [4], there is usually a strong interest in having one number that represents the relative abundance of a gene on a chip. The most common summary measures use a non-model-based robust averaging of measurements in a probe set, such as Affymetrix's MAS5 expression value [5], or a model-based expression index (MBEI [6]) or a log-additive robust-multichip-average (RMA [7]) across chips.

The second crucial aspect of low-level analysis is the control of technical variation between chips, which is introduced by the measurement process during sample preparation, labelling, hybridization, and scanning. Technical variation of this kind and the need for a corrective normalization procedure are not specific to high-density oligonucleotide arrays, but are a general feature of mRNA measurement, e.g. for cDNA microarrays [8], northern-blot analysis or RT-PCR [9]. Numerous procedures have been suggested, differing in their assumptions on what feature of the data remains constant across chips and can therefore be used for normalization [10].

Comparative evaluation of different approaches to low-level analysis has so far been limited to artificial data sets, where differential expression is due to spiked-in RNA or mixtures and dilutions of RNA from different sources [4,10,11]. This has the obvious advantage that the true expression ratios are known (up to experimental error). Consequently, different approaches can be compared in regard to bias (when estimating fold change) and variance (when testing for differential expression). Results so far indicate that there is generally a trade-off between the two, and it seems fair to say that no current method is optimal under all circumstances.

The choice of low-level analysis and especially the choice of normalization have severe impact on the subsequent analysis of the expression data [12]. Given the wide range of methods available, it would be useful to have a method for assessing their relative merits for a concrete data set, without reference to an external spike-in or dilution data set. This is especially true if we have to assume that our data set is not as well behaved as artificial data, either in terms of the percentage of differentially expressed genes or in terms of RNA quality, or both, as for the clinical data set on breast cancer described in the Methods section. In this paper, we propose that by studying coregulation or correlations between random pairs of genes, we can compare different summary measures and assess the effect of different normalization procedures. Our underlying hypothesis is that given a modern large-scale chip covering a large percentage of a species' genome, randomly selected pairs of genes will be *on average *uncorrelated. Note that we do not claim the absence of all biological correlation between genes, but rather that the number of connections between genes in regulatory pathways is small compared to the number of all possible combinations of genes; this argument is given more detail in the Discussion. Consequently, a low-level analysis strategy will be deemed suitable for a given data set, if the resulting normalized expression values are on average uncorrelated for randomly chosen pairs of genes. Lack of correlation is not assessed via formal tests, but by easily adaptable graphical tools that do not rely on stringent conditions for validity.

We proceed as follows: first, we establish relationships between lack of normalization and correlations between randomly selected genes for three important summary measures; then we show that the default normalization schemes associated with these summary measures do remove the correlations to a large degree, but not completely, with varying amounts of residual correlation. We also show that where available, housekeeping gene normalization is inferior to default normalization in removing random correlation, and we relate random correlation to the number of unexpressed genes in the data. We conclude by discussing the results and the underlying assumption of our approach as well as considerations for its practical implementation, and point out both limitations and possible extensions.

## Results

### Lack of normalization is associated with random correlation

We first calculated raw unnormalized MAS5, RMA, and MBEI expression values for the breast cancer, dilution, and spike-in data sets as described in the Methods section. The breast cancer data set is an example of a clinical data set from a real patient population, which is expected to have greater biological variation than the dilution and spike-in data sets. We then computed the Pearson correlation coefficients for 5000 random pairs of probes for each data set.

**Figure 1:**
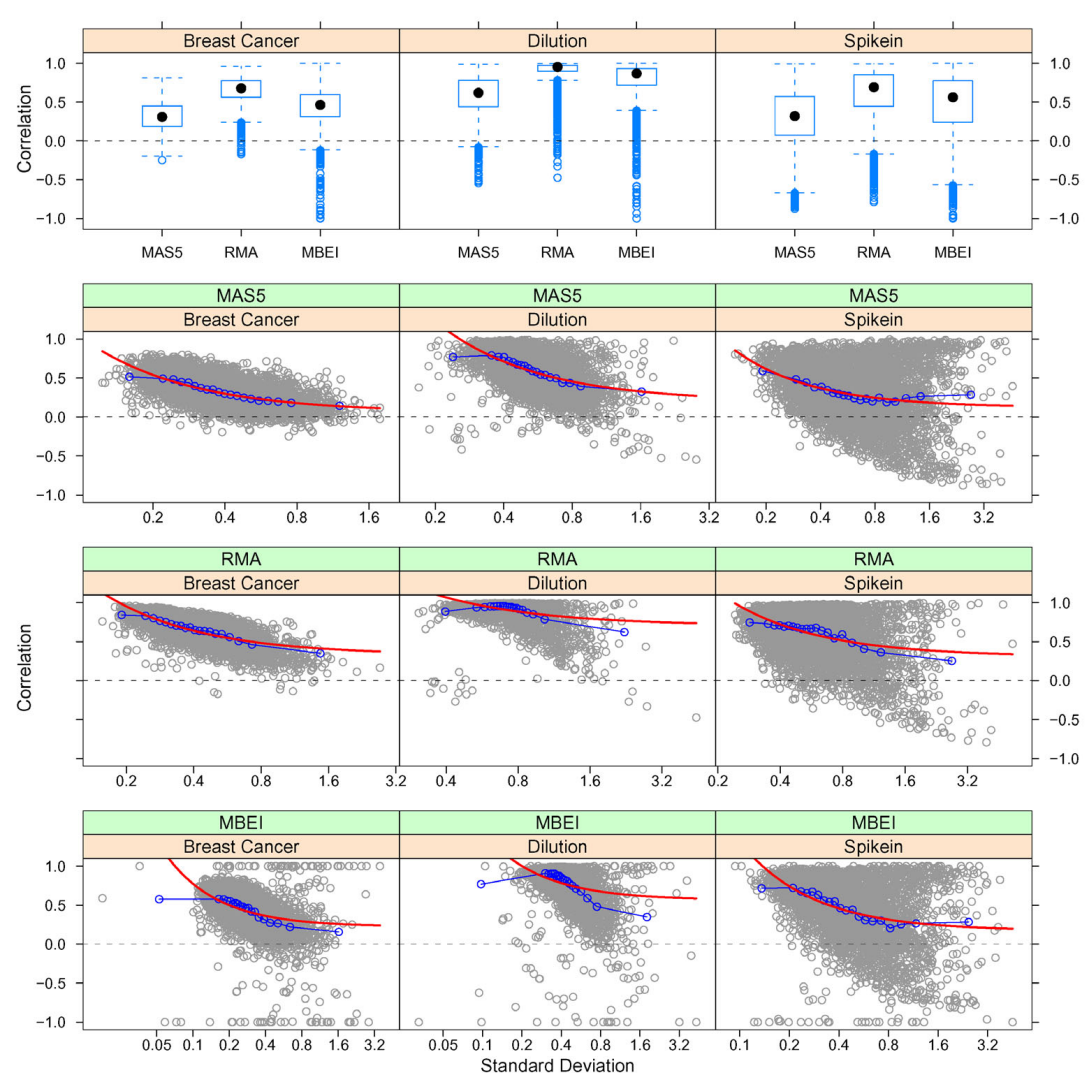
Correlations between the unnormalized expression values of 5000 randomly selected pairs of genes. Top: The distribution of the correlation coefficients is centered far away from zero for all expression measures and data sets. Bottom: Scatterplots of the correlations versus the product of the standard deviations of the genes for the randomly selected pairs. Local mean correlations are shown in blue and indicate that correlations decrease with variability. Shown in red is a simple model for lack of normalization that was fitted to the data.

As shown in the upper part of Figure 1, the distributions of the correlation coefficients are centered far away from zero for each data set and expression measure. There is clearly a large amount of excess correlation that is unrelated to biological relationships between genes. The similarity of expression between random pairs of genes across chips is due to technical differences between chips which have not been normalized out. This is a striking example of statistical confounding, where genes are apparently correlated for some underlying non-biological reason.

We have also found that the technical correlation between genes is inversely related to the variability of the genes involved. This can be seen in the lower part of Figure 1, where the correlations between the random pairs are plotted against the product of their standard deviations: the average correlation (shown in blue) is highest for genes with small standard deviations and decreases with increasing variability. This fits well with what we would expect from assuming a simple additive chip effect as the source of chip-to-chip variation; even though this is certainly an oversimplification, the corresponding model fits the general shape of the data well enough (shown as red line in Figure 1; see Methods).

### Default normalization removes excess correlation

**Figure 2:**
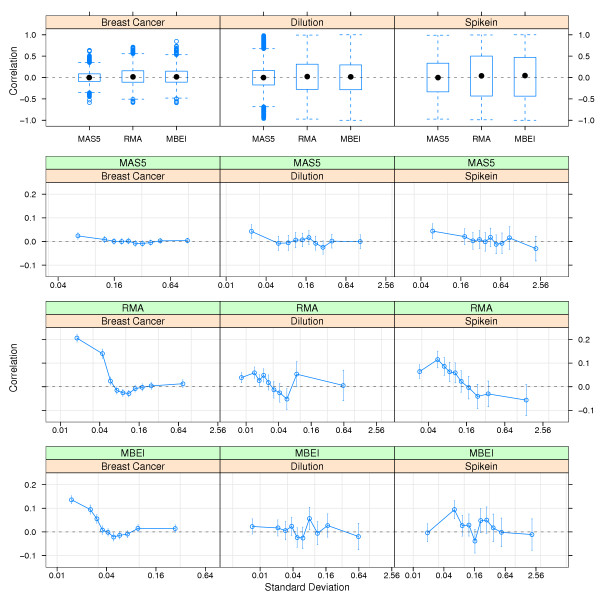
Correlations between the normalized expression values of the 5000 randomly selected pairs of genes. The most commonly used normalization procedure was chosen for each expression measure: global mean normalization for MAS5, quantile normalization for RMA, and invariant set normlization for MBEI, see Methods. Top: The distributions of the correlation coefficients are now centered at zero for all expression measures and data sets. Bottom: Summary curves plotting mean correlations versus median product of standard deviations for pairs of genes in non-overlapping intervals along the horizontal axis; 95% confidence intervals are shown as vertical bars. Normalization has removed most of the technical correlation (as seen in the reduced vertical scale compared to Figure 1), but there are still systematic relationships between correlations and standard deviations for RMA and MBEI, which are most pronounced for the Breast Cancer data set.

We calculated the same expression measures for the same data sets as above, but applied the default normalization procedure suggested for each expression measure: for MAS5 expression values, we normalized to the global mean within each array, for RMA values, we applied the quantile normalization, for MBEI we applied the invariant set normalization, see Methods. The upper part of Figure 2 shows that in all cases, the default normalization step was sufficient to remove excess correlation and center the distribution of the correlation coefficients at zero.

In the following, we will refer to unwanted correlation artifacts after normalization as residual correlation. Although we observed no residual correlation for the whole set of genes, there was no guarantee that this would hold for certain subsets of genes: an ideal normalization should remove the residual correlation for any sufficiently large subset of genes. Therefore, we investigated the pattern of correlations for pairs of genes with different intensity and variability across chips.

### Genes with low variability are poorly normalized by RMA and MBEI

We previously described the systematic inverse relationship between correlation and variability. Although the default normalizations strongly reduced the scale of this correlation for all three expression measures, we still found a significant relationship between correlations and variability for RMA and MBEI, especially for the breast cancer data. The lower part of Figure 2 shows the average correlations between genes grouped by the product of their standard deviations; this is the same summary line as in Figure 1, but without plotting the individual points contributing to it. The residual correlations were smaller than before normalization, but the approximate confidence intervals show them to be highly significant. The shape of the relationship also changed and did no longer follow any simple model.

We found that the residual correlations were both absolutely larger and more significant for RMA than for MAS5. For MAS5, only the subset of genes with the lowest variability showed significant correlation, all of it positive and less than 0.05. In contrast, for RMA and MBEI, several of the low-variability classes showed significant positive correlation, up to 0.2 for the breast cancer data set. In addition, we observed small but significant negative correlations for genes in the middle range of variability for the breast cancer and dilution data.

Thus the analysis shows that RMA and MBEI do not provide properly normalized expression values for genes with low variability, particularly for the clinical data. We will explain this pattern later in terms of absence and intensity of genes.

### Normalization on housekeeping genes fails to remove excess correlation

The HGU133A chips that were used for the breast cancer study contain 100 probes for generic housekeeping genes, whose expression is assumed to be constant on average for most or all experimental conditions. Consequently, it has been suggested to use these housekeeping genes for normalization, by adjusting the expression level on each chip so that the average expression of the housekeeping genes is constant across chips (see Methods). To date, there is no convincing evidence whether this method actually works or not, and it seems that some research groups are using it.

**Figure 3:**
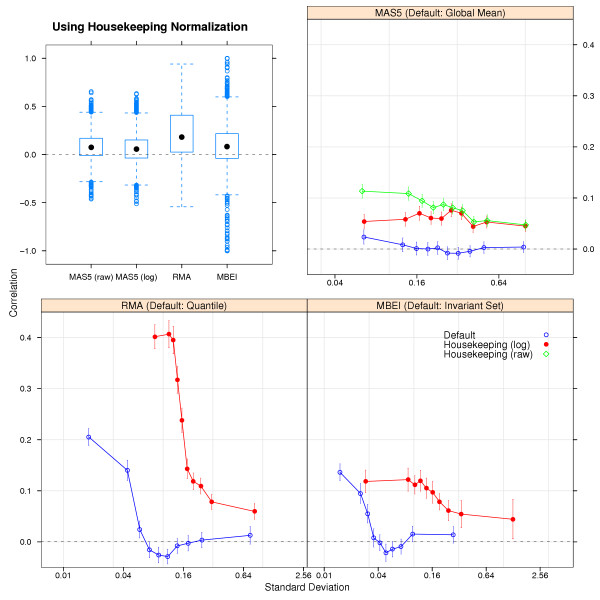
Correlations for the Breast Cancer data set normalized using a predefined set of 100 houskeeping genes. The MAS5 expression values were normalized both before and after taking the logarithm, corresponding to MAS5 (raw) and MAS5 (log). The RMA and MBEI expression values were normalized after caculating the logarithmized expression values, see Methods. Top left: The distributions of the correlation coeffcients are not centered at zero. Top right and bottom: Mean correlations versus median product of standard deviations are shown as in Figure 2, comparing the residual correlation for the housekeeping normalization and the default procedure for each expression measure (as indicated in the legends). Housekeeping normalization does much worse than the default in removing the systematic relationships. In case of the MAS5 values, both variants do worse than the default, and the normalization of the raw (un-logged) values doing worse than that of the logarithmized values.

The correlation test given in Figure 3 shows that for the MAS5, RMA and MBEI methods of computing expression values, the housekeeping gene normalization failed to remove the excess correlation. There was nonzero average correlation over all genes, indicating a general failure of normalization. The systematic inverse relationship between correlation and variability were at higher levels throughout the range of variability compared to the default normalizations. The failure of housekeeping gene normalization was particularly severe for RMA.

**Figure 4:**
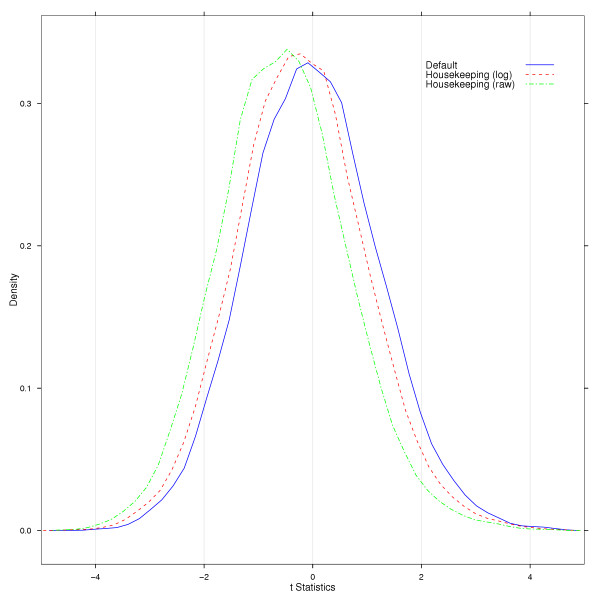
Distributions of t-statistics calculated from differently normalized MAS5 expression values in the Breast Cancer data. The normalization procedures applied were the same as in Figure 3: global mean as default and housekeeping normalization before and after taking logarithms (raw and log respectively in the legend.) The t-statistics compare expression values between users and non-users of hormone replacement therapy (HRT) in the sample. The density curves show the smoothed histograms of the test statistics for all 22283 probe sets on the chip. The default curve is centered at zero, indicating balanced up- and down-regulation in reaction to HRT, whereas the curves for the housekeeping-normalized data are shifted to the left, indicating a massive down-regulation of thousands of genes in HRT users, which is biologically much less plausible. This effect is more pronounced for the raw than for the logged normalization, same as with the residual correlations shown in Figure 3.

Note that even if the amount of residual correlation shown in Figure 3 for MAS5 housekeeping-genenormalized values looks small, the impact on the subsequent high-level analysis can be serious. Figure 4 shows the distribution of 22283 gene-wise t-statistics for the housekeeping-normalized and the global-mean-normalized breast cancer data. Each t-statistic compares the mean expression level between (a) postmenopausal women who are users of hormone replacement therapy (HRT) versus (b) those who are not; see (for personal communications see Hall P, Ploner A, Bjöhle J et al.). The t-statistics for the housekeeping-normalized values are globally shifted below zero, indicating a genome-wide down-regulation of thousands of genes. In contrast, the t-statistics based on global-mean-normalized values are centered around zero, suggesting a much less pronounced difference between HRT users and non-users. In this example, the global-mean-normalized results are biologically much more plausible.

### Absent genes are poorly normalized by RMA and MBEI

In each tissue, only a limited number of genes will be expressed in quantities above the detection limit, usually much fewer than the number of genes available on modern large-scale chips. The purpose of pairing PM and MM probes is to detect which genes are reliably expressed (present genes), and for which genes the observed intensities are dominated by technical and biological noise (absent genes). The most common method of classifying genes as either present or absent is based a non-parametric test for the PM/MM pairs (Affymetrix's detection calls [13]).

There is currently no consensus on how to use these detection calls. All methods report expression values of all genes including the absent genes, so in principle the analyst might ignore the issue of absent genes and treat all genes as present. Intuitively the absent genes will be measured with a lot of noise, but will they be properly normalized, i.e., will the measurements be unbiased?

**Figure 5:**
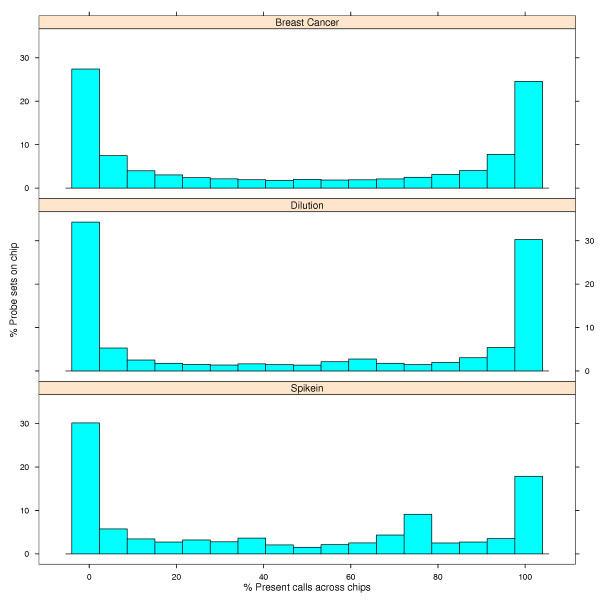
Percentage of Affymetrix present calls across all chips for all three data sets. For each probe set, the percentage of present calls across all chips in its data set was calculated. The histograms show the frequencies of these percentages. For each data set, about 30% of all probes are absent on all chips, and between 20 and 30% are present on all chips, with the balance equally distributed between the extremes.

In order to study the success of normalization of measured expression of absent genes, we classified all genes as either present or absent based on Affymetrix's present calls (see Methods). For all data sets, genes were most frequently either completely absent or completely present across all chips (Figure 5).

**Figure 6:**
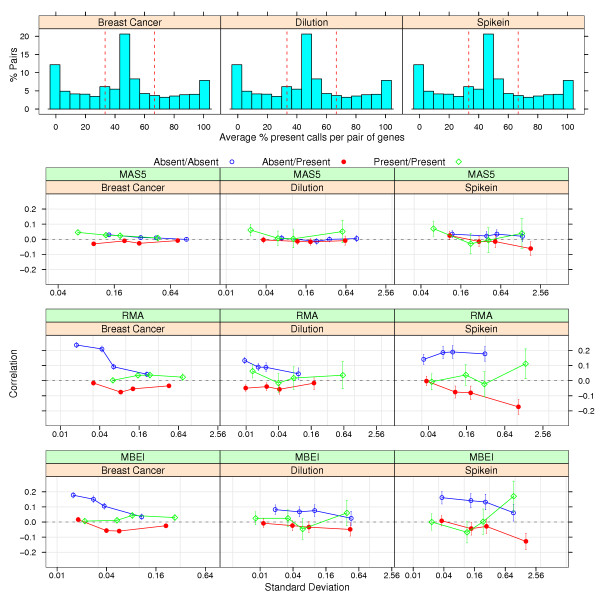
Correlations of randomly sampled pairs of genes by percentage of Affymetrix present calls. Top: Histograms of the average number of present calls for the 5000 pairs of genes, showing three peaks at the ends and in the middle of the range. The red lines seperate the peaks and indicate the grouping of the percentages used in the bottom part of the figure. Looking at Figure 5, we identify the groups with pairs of genes where both genes are mostly absent (left peak), where both genes are mostly present (right peak), and where one gene is mostly absent and the other mostly present (central peak). Bottom: Summary curves for the correlations as a function of the product of standard deviations. The different curves correspond to the three groups of pairs of genes as identified in the histograms above. Note how correlations are consistently positive for absent/absent pairs and consistently negative for absent/present pairs in case of RMA and MBEI. Only the present/present pairs appear to be reasonably uncorrelated throughout.

Consequently, the pairs of genes in our random samples could naturally be divided into three classes: those averaging few or no present calls between them, those averaging almost a 100% present calls, and those averaging around 50% present calls (upper part of Figure 6). These classes correspond naturally to pairs where both genes were mostly absent, or both mostly present, or where one was mostly absent and the other mostly present; by cutting at 33% and 67% average present calls as indicated in the histograms in the upper part of Figure 6, we managed to separate these groups evenly.

To provide more information, the average correlation for each subset was again plotted against variability; see lower half of Figure 6. Generally, the average correlation was highest for pairs of absent genes, indicating failure of normalization of measured expression of absent genes. This was most serious for RMA: excess correlations were consistently and strongly positive for absent pairs and negative for absent/present pairs for all data sets. Only for present pairs, correlations were mostly non-significant and small in absolute value. Correlations for MAS5 were throughout smaller and less significant, with no clear pattern between the three groups of pairs. MBEI showed the same pattern as RMA, though somewhat weaker.

**Figure 7:**
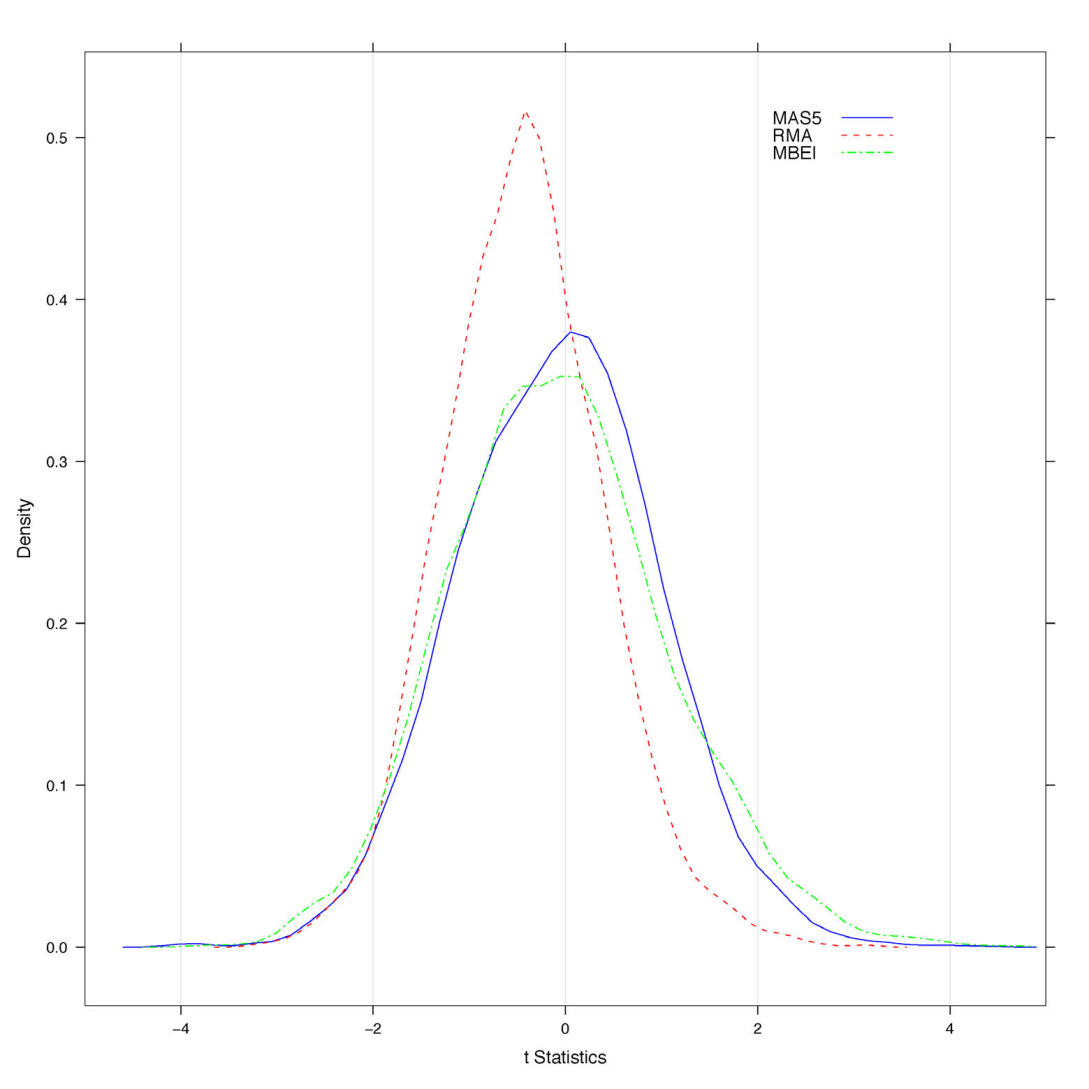
Distributions of t-statistics calculated from different expression measures for genes that are completely absent in the Breast Cancer data. The same default normalization procedures as in Figure 2 were applied. The t-statistics compare expression values between users and non-users of hormone replacement therapy (HRT) in the sample as in Figure 4. The density curves show the smoothed histograms of the test statistics for 4371 probe sets that had absent calls on all 159 chips. The MAS5 curve is centered at zero and close to a standard normal distribution expected approximately if no gene is differentially expressed between HRT users and non-users. The RMA curve is strongly shifted to the left, indicating wide-spread down-regulation of numerous absent genes in non-users; the MBEI curve shows moderate asymmetry and heavy tails at both sides, indicating more moderate, but still common gene regulation. Given the low signal quality of these genes that were classified as absent throughout the data set, small or no detectable regulation effects seem biologically most plausible.

This result implies that, at least in case of RMA and MBEI, measured expressions of absent genes were poorly normalized, so analyses of absent genes should be avoided or at least viewed with caution. This interpretation is supported by Figure 7, which shows the distribution of t-statistics comparing HRT-users and non-users as above, but only for genes that were not detected (absent) on all 159 chips (*n *= 4371); the distributions for MBEI and especially RMA indicate strong and wide-spread regulatory effects of HRT, which seems biologically implausible, especially for genes measured at the detection limit throughout the data set.

While the absence or presence of a gene could be assessed via other potential quality control measures, the Affymetrix detection call seems to provide useful information for gene filtering.

Note that the summary curves of mean correlation shown in Figure 2 are the weighted means of the curves by presence status shown in Figure 6. We can, for example, explain that the high correlations at low variability for RMA in Figure 2 are mainly due to absent/absent pairs in the expression data. The slight negative dip for genes at the middle range of variability in Figure 2 is the effect of an incomplete cancelation between the positive correlations for absent/absent pairs and the negative correlations for absent/present pairs in this range.

### Residual correlation is only weakly related to the expression level of genes

Detection of a gene is trivially related to the relative abundance of its mRNA in the sample. Thus, genes that are expressed at the lower end of the detection range are much more likely to be absent. This might indicate that the relationship between the absence/presence of genes and their residual correlation is in fact due to their difference in abundance, and that by focusing on genes with a minimum expression level, we could avoid residual correlation altogether.

**Figure 8:**
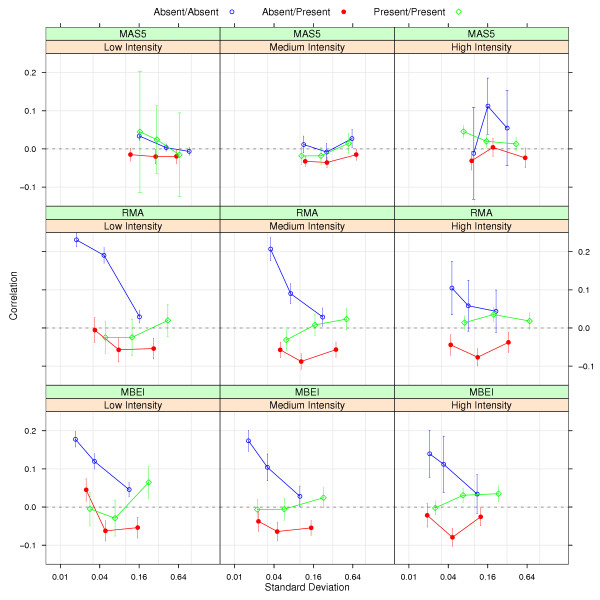
Correlations of the randomly sampled pairs of genes by percentage of present calls and mean intensity. The summary curves show the average correlation of the pairs of genes sampled from the breast cancer data. The differently colored curves correspond to the groups of absent/absent, absent/present and present/present pairs that have been defined in the text and are shown in Figure 6. Additionally, the pairs of genes have been arranged by their average mean intensity across chips: the range of average intensities was split into three intervals with an equal number of pairs, from the lowest third on the left to the highest third on the right. Grouping the pairs by their absence/presence status yields distinctly different patterns of correlations for RMA and MBEI, these patterns are however highly consistent between the intensities.

Figure 8 shows that this is not the case: when plotting correlations against standard deviations grouped by intensity in the breast cancer data, we found that the pattern of correlation depends more on the percentage of present calls than on the intensity level. The pattern we saw previously in Figure 6 was observed at different levels of intensity: (i) pairs where both genes are mostly absent tend to be positively correlated, (ii) pairs with one gene mostly absent and one gene mostly present tend to be negatively correlated, and (iii) genes where both partners are mostly present tend to be almost uncorrelated. This pattern is most pronounced at low and medium intensities, and it is stronger for RMA and MBEI, but it is consistently seen, also at high intensities and for MAS5 values.

In summary it seems strongly preferable to define a gene filter according to absent/present calls than according to the gene intensity levels.

Note that correlation between the intensity and presence of genes is reflected by the number of pairs that contribute to each curve in Figure 8: there were relatively more present/present gene pairs and less absent/absent pairs at high intensities, and vice versa for low intensities; curves with lower pair counts have correspondingly wider confidence intervals.

### Filtering out absent genes reduces residual correlation

**Figure 9:**
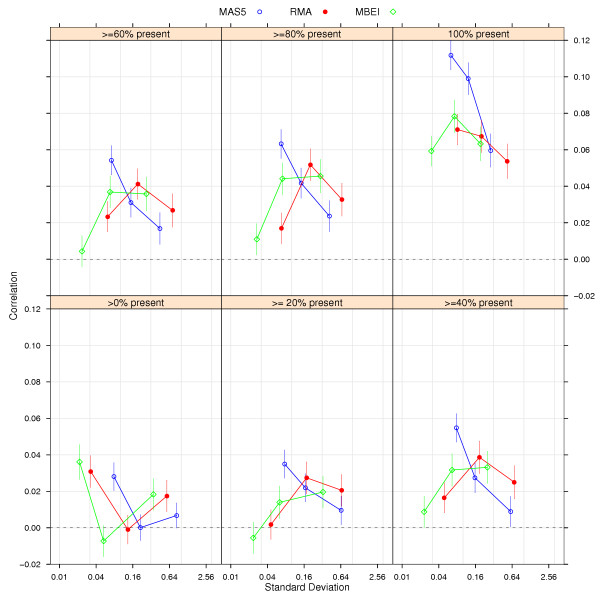
Correlations of pairs of genes sampled from subsets with an increasing minimum percentage of present calls for probe sets. From the breast cancer data, 5000 pairs of genes were randomly sampled from the subset of probe sets that were present on at least one of the chips, the subset of probe sets that were present on at least 20% of the chips etc., finally from the subset of probe sets present on all chips. The three curves represent the three expression measures. Excluding the probe sets that are always absent already reduces the residual correlation considerably, excluding the probe sets that are more than 80% absent breaks the pattern of highest correlation at smallest variability for RMA and MBEI seen in Figures 2, 3, 6, and 7. Using only probe sets that are always present however leads to a notable increase in residual correlation, especially for MAS5.

Figure 9 demonstrates for the breast cancer data how the filtering of genes with a large number of absent calls can reduce residual correlation for normalized expression values. In this case, the 5000 pairs of genes were randomly sampled from subsets of genes with an increasing percentage of present calls. Already by excluding genes that are always absent, the level of systematic correlation was reduced below 0.04 for all expression measures, though the pattern of positive correlations for genes with low variability was still present; by considering only genes with at least 20% present calls, we found that this pattern is reversed for RMA and MBEI, but not for MAS5. Further restrictions did not change this pattern, but increased the absolute level of residual correlation.

## Discussion

### The assumption of zero correlation

**Figure 10:**
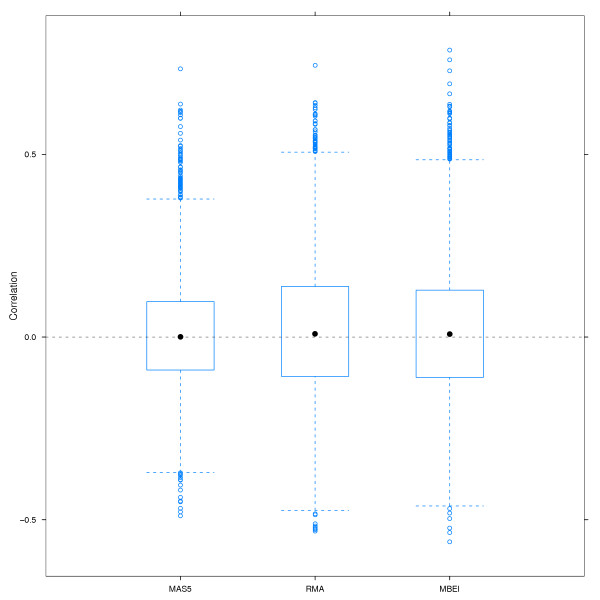
Correlations for 5000 random pairs of probe sets selected from KEGG pathways. Pairs were sampled from the subset of probe sets in the breast cancer data that appear in at least one KEGG pathway. Even for this smaller (*n *= 3137) and well-connected subset, the correlations are centered around zero.

As some genes are connected in biochemical pathways, the hypothesis that random pairs of genes will be on average uncoregulated or uncorrelated seems counterintuitive, but it is really a question of scale. For a moderately large chip of 10000 probe sets, there are about 50 million possible pairwise correlations, the huge majority of which will be extremely unlikely to be biological. Any random sample of probe set pairs will contain only a small percentage of pairs representing an unequivocal biological relationship, and additionally, negative and positive correlations will tend to cancel each other out during averaging. We can demonstrate this for the breast cancer data set. On the Affymetrix HGU133A chip, we find represented 124 KEGG pathways, organising 3137 probe sets or 14% of all probe sets on the chip ([14], build 2004/03). This constitutes an as highly-organised subset of the genome as we can currently hope to select, with numerous probe sets appearing in multiple pathways, thereby establishing numerous cross-correlations between pathways. Figure 10 shows the boxplots of correlations for 5000 randomly selected pairs of genes from this subset, firmly centered at zero for all three expression measures. So even for this special subset of many coregulated genes, the average correlation of a random pair of genes is zero.

### The simple model of lack of normalization

The model described in the Methods section only assumes differences in mean intensity between chips. This corresponds to the simple global mean normalization commonly used for the MAS5 expression values. Figure 1 confirms that this model (shown in red) describes the average behavior of the correlations (shown in blue) adequately for all data sets, suggesting that global mean normalization is indeed suitable for MAS5 data.

Apart from MAS5, the model fits adequately only for the RMA-based correlations in the breast cancer data, suggesting that global mean normalization on the probe-set level may be attempted in this case, but that it is not generally suitable for RMA and MBEI data. Still, Figure 1 shows that correlations decrease systematically with the variability of the gene pairs for all expression measures, and it may be possible to describe this relationship by extending the simple model, e.g. by allowing the array effect *θ *in Equation 1 to be correlated with the gene effects *ψ*_*i*_.

### The bad performance of housekeeping genes

The use of housekeeping genes seems reasonable when studying a small number of genes under controlled experimental settings, or where the choice of one or several housekeeping genes can be motivated biologically. For the breast cancer data, which was collected in a real clinical setting, where samples are both genetically heterogenous and potentially genomically unstable, it is much harder to believe in the common expression of housekeeping genes. Therefore we argue that the failure of housekeeping normalization in this example is not due to the procedure per se, but to our inability to identify a suitable set of housekeeping genes, and the use of the generic set of genes suggested by the chip manufacturer. Even for northern-blot analysis and RT-PCR, where housekeeping normalization is the default, an uncritical use of housekeeping genes has been shown to lead to unacceptable results [9].

### Comparison of MAS5, RMA and MBEI

It has been suggested that the generally much lower variability of RMA and MBEI for low-intensity probe sets is a clear advantage of these model-based expression measures over the simpler MAS5 [7,10]. Our results however indicate that this low variability may well be misleading: RMA and MBEI values for absent probe sets, which constitute the vast majority of low-intensity probe sets, show the strongest residual correlation. This indicates that RMA and MBEI values for low-intensity probe sets that are reported without regard for their absence/presence status will be compromised by lack of normalization (Figures 6 and 8). It seems therefore that RMA and MBEI estimate expression of low-abundance genes in a biased, but very precise manner. Minimizing variability as much as possible only makes sense for unbiased estimato rs: if the variability of the estimate becomes small relative to the bias, we get a dangerous sense of confidence in an estimate that is not quite what we think it is. In the same way, the large variability of the MAS5 values at low intensities may well hide an amount of bias comparable to that of RMA and MBEI: as long as the variability of MAS5 is large compared to the bias, we will not be lead to make inappropriate conclusions based on possibly biased estimates; in that sense the MAS5 estimates for low-intensity genes are more honest and better normalized than the corresponding RMA and MBEI values. It is interesting to note that Bolstad et al. have already described the choice between different low-level approaches in terms of bias (when estimating fold change between conditions) and variance (when testing for differential expression between conditions) [10]. Our results suggest that a) the same trade-off applies when looking directly at the expression values, instead of comparing aggregated fold changes and test statistics between different biological conditions, and b) the trade-off is more disadvantageous for the model-based expression measures than generally thought.

The underlying lack of normalization of RMA and MBEI for absent genes could be due to the computation of the expression values, or the normalization step, or a combination thereof. Preliminary results (not shown) indicate that the first step, the summarization of the individual probe intensities through the expression measure, seems to be responsible in both cases. If this can be confirmed, a possible explanation would be that the models used (log-linear for RMA and and multiplicative for MBEI) may not be appropriate for absent genes (but see also below).

### Improving low-level analysis

In a recent paper, Choe et al. have evaluated the performance of a wide range of low-level analysis methods and test procedures in detecting differential expression in a carefully constructed spike-in data set [15]. They report 70% sensitivity at 10% false discovery rate for their top-ranking combinations clearly there is still ample room for improvement in current low-level methodology. We want to outline here shortly how our approach could be used to guide this effort.

**Figure 11:**
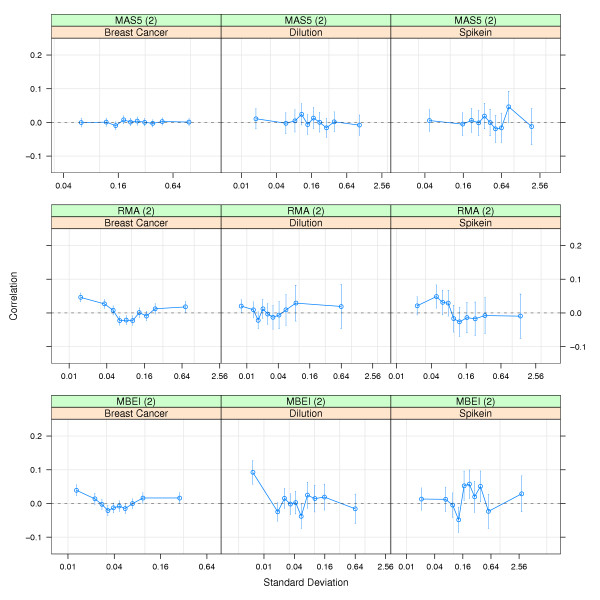
Correlations after renormalizing the expression measures using the pairwise loess normalization on the probe set level. Except for the second normalization step, this is identical to the lower half of Figure 2, including the scaling of the axis to facilitate the comparison. The MAS5 values appear now well normalized throughout, whereas RMA and MBEI still show a similar pattern of residual correlation, although on a clearly reduced level.

The authors of [15] found that an additional second step of normalization on the probe-set level improved the performance of MAS5, RMA, and MBEI in detecting differential expression (indeed, MAS5 with the second round of normalization was one of the top-ranking combinations). We have applied the same renormalization to our data sets (see Methods for details), the results are shown in Figure 11.

We found that renormalization reduced residual correlation for all data sets and all expression measures. Indeed, for MAS5 the correlations are not significantly different from zero at any lag, indicating perfect normalization as measured by our criterion. RMA and MBEI show strongly reduced levels of residual correlation, but are still well above the levels of the original MAS5 as seen in Figure 2.

It is interesting that the ranking of the original and renormalized expression measures in terms of normalization quality (i.e. renormalized MAS5 is best, followed by the original MAS5, followed by renormalized RMA and MBEI, followed by the original RMA and MBEI) corresponds closely to the ranking by performance in detecting differential expression found by Choe et al. ([15], Figure 7f). This suggests that the lack of normalization that our method is able to measure is indeed relevant for the ability to detect regulated genes.

Additionally, Figure 11 gives an indication of how the newly renormalized expression measures may be further improved. E.g. for the renormalized MAS5, there is clearly little need to work on the normalization aspect; modifications of the expression measure could instead aim at reducing the variability of MAS5 values, possibly by using the information in the MM probes as weights in the summary measure.

Renormalized RMA and MBEI on the other hand still suffer from insufficient normalization; as we perform already normalization steps on both the probe and the probe set level, it seems promising to focus on the intermediate steps like the fitting of the multi-chip model and to study whether these steps are prone to systematic biases.

### Limitations

The only condition for using the correlation test is a fairly large chip, with probes covering a wide range of the genome under study. For chips that are designed to study only a few related pathways or highly specialized tissues with only a couple of hundred probe sets, the zero correlation assumption may not hold, because the genes from which we want to sample randomly have already been pre-selected by the chip design. The example of the KEGG probe sets on the U133A chip suggests though that several thousand probe sets organized in a hundred and some pathways is a safe size.

It should be pointed out that this approach is not limited to high-density oligonucleotide chips. The same argument for between-chip normalization holds in principle for cDNA or any other two-color microarray system, although the usual intensity-based normalization between dye channels on the same chip simplifies the situation somewhat [16].

## Conclusion

We have presented a simple graphical method for assessing the quality of low-level analysis of oligonucleotide array expression data. The main advantage of our approach lies in the fact that we do not make use of external reference data, but instead exploit the internal correlation structure of large expression data sets. This allows us to select, evaluate, and modify low-level procedures for specific data sets. In order to demonstrate the use of and usefulness of our approach, we have applied it to three large data sets and three widely used low-level methods (MAS5, RMA, MBEI). We found a number of interesting results: a) For a large breast cancer data set, normalizing to housekeeping genes does not work at all, regardless of expression measure; b) normalization quality for all three data sets and all three expression measures is closer related to the absence/presence status of a probe set than to its intensity level; c) RMA and MBEI normalize absent probe sets poorly for all three data sets; d) removing pre-dominantly absent probe sets improves normalization for all data sets and all expression measures. The cutoff percentage of absent calls for a probe set to be included in the analysis can be chosen based on our graphical criterion. We have also evaluated the effect of a second round of normalization on the probe set level data. We found that this improved normalization significantly for all three data sets, in a manner consistent with the observed improvements in the detection of gene regulation [15].

## Methods

### Data

We used three data sets, two of which are publicly available from GeneLogic [17]. (1) The dilution data set is a collection of 75 HGU95Av2 chips, on which RNA from two different sources (liver and nervous system) was hybridized in different concentrations and mixture ratios. (2) The spike-in data set consists of 94 HGU95Avl chips, for which eleven bacterial cRNA fragments were added in different concentrations and combinations to a base sample from an AML cell line. Both of these data sets have been widely used for assessing normalization methods and expression measures [4,7,10].

In contrast the RNA for the third data set was extracted from tumor tissue collected from a population-based breast cancer cohort at Karolinska Hospital, Stockholm. After processing the RNA, several quality control steps, and screening the patients on medical criteria resulted in data from 159 HGU133A chips. Details on data preparation, patient selection, and the definition of clinical parameters like hormone replacement therapy are given in [for personal communications see Hall P, Ploner A, Bjöhle J et al.].

### Expression measures and normalization methods

MAS5 expression values were computed as described in [5]. We used global mean normalization for the logged expression values as default, assuming that the mean across the logged expression values of all probes should be constant across all chips, and adjusting the level of each chip by adding a corrective constant to all probes. This is roughly equivalent to using the standard Affymetrix scaling factors on the raw data, but estimation of the corrective term on the log-scale has been found to be less variable [18]. The corrected log values were used for the analysis.

For RMA, the individual PM probe values were background-corrected and quantilenormalized before computing the expression values, as described in [7].

The computation of MBEI expression values followed [6]: PM and MM values were normalized separately to a baseline array of average PM and MM intensities. The baseline array was obtained via smoothing an empirically identified set of rank-invariant probes. A multiplicative model was fitted to the difference between normalized PM and MM values. Expression values were logged, with non-positive values set to missing.

Housekeeping gene normalization was based on the probes with suffix 2000_ on the HGU133A chip. The same principle as with the global mean normalization was employed, except that the correction constant was based on the average of the housekeeping genes. For the MAS5 values, two variants were considered: a) multiplicative correction (scaling factor) of the un-logarithmized expression values, referred to as 'raw housekeeping' in the legends for Figures 3 and 4b) additive correction of the logarithmized expression values, as for the global mean normalization, referred to as ' log housekeeping' in the figure legends. Housekeeping normalization for RMA and MBEI was done additively for the logarithmized expression values calculated from the unnormalized probe data; therefore, these are also addressed as 'log housekeeping' values in Figure 3.

The renormalization of the expression measures described in the Discussion was performed as in [15], using the iterated pairwise intensity-based normalization via smoothing loess curves described previously in [10]. The renormalization was performed at the probe set level on the expression measures computed and normalized as described above; correspondingly, renormalized RMA and MBE values have been normalized on both the probe level (originally) and the probe set level (second round), whereas MAS5 has been normalized twice on the probe set level.

All computations were done using the open source statistical software package R [19] and the package affy of the Bioconductor project [20].

### Calculation of correlations and summary curves

For each data set, we randomly selected 5000 pairs of probe sets from the collection of probe sets available on the different chip types. For each pair, we computed the Pearson correlation coefficient between the two probe sets across all chips in the underlying data set, resulting in a random sample of 5000 correlation coefficients. At the same time, we calculated for each pair of probe sets the product of the two standard deviations across all chips in the data set; the scatter plots in the lower part of Figure 1 show the resulting 5000 pairs of (product of standard deviation, correlation).

The summary curves shown on top (like in Figure 1) or instead (all other Figures) of the point scatter describe the average behaviour of the scattered cloud. They were produced by taking the range of values for the product of the standard deviations in the sample and splitting it into intervals containing an equal number of observations, typically around 500. For each interval, the mean of the correlations was plotted against the median of the product of standard deviations. The 95% confidence intervals of the means shown from Figure 2 onwards were computed based on normal approximation.

### A simple model for lack of normalization

We assume as experimental unit one microarray chip with the associated samples from the biological population under study. Each chip yields observations *y*_*i *_for *i *= 1 ... *n *genes specified by the array design. We can write this as a random variable

*Y*_*i *_= *θ *+ *ψ*_*i *_+ *ε*_*i *_    (1)

where *θ *is a random array effect, *ψ*_*i *_is a random gene effect, and *ε*_*i *_is the gene-specific measurement error. Note that this assumes random gene effects only in so far as we sample from the population, and we do not specify any treatment or experimental structure.

We assume that the random components are independent, and that the errors have expectation zero. The covariance between the observable expression values for two genes then simplifies to



i.e. the covariance between the unobservable 'real' gene expressions plus the variance of the array effect. Let's designate the variance of any *Y*_*i *_as . Now the correlations between the observable expression values for two genes can be written as



The first term is the contribution of the array effect to the correlation, which is the source of the correlation artifact. The second term varies across all possible pairs of genes, and we expect it to have zero average. Thus we get the inverse relationship



We can investigate this empirically. Given a set of microarrays, we can take a sample of random pairs of genes (*i*, *j*), then calculate their correlations *r*_*ij *_and standard deviations *s*_*i *_and *s*_*j*_. Under our hypothesis of zero average correlation and assuming that our simple model holds, the underlying pattern in the plot of (*s*_*i*_*s*_*j*_, *r*_*ij*_) should follow this inverse relationship.
